# Correction of forearm deformities in BPBP

**DOI:** 10.1186/1753-6561-9-S3-A16

**Published:** 2015-05-19

**Authors:** Praveen Bhardwaj

**Affiliations:** 1 Ganga Hospital, Coimbatore, 641043, India

## 

In patients with Brachial Plexus Birth Palsy (BPBP) both pronation and supination deformities can happen depending on the extent of injury, pattern of recovery and the resulting muscle imbalance. The main aim of the corrective surgery is to improve function but improving appearance of the limb is also an important consideration.

Pronation posture is more important for bimanual and table top activities while the supination position is required for eating and worshiping activities in some cultures. In the limb which is being used for eating, supination restoration procedures are indicated. Supination deformity is cosmetically bad and has been termed as- “beggar's hand” or “unshakable hand”. It also places the hand in less functional position and results in disability more than that would be expected for the existing muscle power in the hand. Correction of the deformity places the hand in a cosmetic position and also improves the function of the hand.

## Restoration of forearm supination

The technique of correction depends on the status of the possible donors, the associated deficits requiring correction and presence of passive forearm rotation. Tendon transfer procedures have the advantage of maintaining movement in opposite direction but a bony procedure e.g. forearm osteotomy decreases the rotation in the opposite direction. In patients with a fixed deformity, radius supination osteotomy is the only option. For stiff terminal movements, interosseous membrane release can improve the passive rotation range and allow us to perform a tendon transfer surgery. The tendon transfer options include- pronator teres rerouting; flexor carpi ulnaris to split brachioradialis transfer and brachioradialis rerouting. All the procedures work well in an appropriately selected patients. Flexor carpi ulnaris transfer is the strongest but it can only be used when this muscle is not needed to restore any other more useful hand function.

## Restoration of forearm pronation

Here again the technique of correction depends on the status of the possible donors, the associated deficits requiring correction and presence of passive forearm rotation. Tendon transfer procedures have advantage of maintaining opposite movement but the bony procedure e.g. forearm osteotomy decreases rotation on the opposite side. In patients with a fixed deformity, radius pronation osteotomy is the only option. The prerequisite is anti-gravity wrist extension (MRC grade 3). If absent, this has to be restored by tendon transfer either at the same sitting or before the osteotomy procedure lest a wrist drop posture results which is more disabling. If there are no donors to restore wrist extension, the wrist can be fused in a functional position and the forearm positioned in pronation with a combined osteotomy procedure.

The tendon transfer options include- biceps rerouting and Brachioradialis rerouting. Biceps rerouting is a good procedure but it can be done only when the tricpes power is more than grade 3 or else there is a risk of increasing the flexion deformity at the elbow in these patients. Brachioradialis rerouting can then be done in such patients with satisfactory results.

